# Development of a mechanistic dengue simulation model for Guangzhou

**DOI:** 10.1017/S095026881900030X

**Published:** 2019-03-01

**Authors:** G. Mincham, K. L. Baldock, H. Rozilawati, C. R. Williams

**Affiliations:** 1 School of Health Sciences, University of South Australia, Adelaide, SA 5001, Australia; 2 Medical Entomology Unit, Infectious Diseases Research Centre, Institute for Medical Research, Ministry of Health Malaysia, 50588 Kuala Lumpur, Malaysia; 3 School of Pharmacy and Medical Sciences, University of South Australia, Adelaide, SA 5001, Australia

**Keywords:** *Aedes albopictus*, dengue fever, intervention strategies, simulation modelling

## Abstract

Dengue infection in China has increased dramatically in recent years. Guangdong province (main city Guangzhou) accounted for more than 94% of all dengue cases in the 2014 outbreak. Currently, there is no existing effective vaccine and most efforts of control are focused on the vector itself. This study aimed to evaluate different dengue management strategies in a region where this disease is emerging. This work was done by establishing a dengue simulation model for Guangzhou to enable the testing of control strategies aimed at vector control and vaccination. For that purpose, the computer-based dengue simulation model (DENSiM) together with the Container-Inhabiting Mosquito Simulation Model (CIMSiM) has been used to create a working dengue simulation model for the city of Guangzhou. In order to achieve the best model fit against historical surveillance data, virus introduction scenarios were run and then matched against the actual dengue surveillance data. The simulation model was able to predict retrospective outbreaks with a sensitivity of 0.18 and a specificity of 0.98. This new parameterisation can now be used to evaluate the potential impact of different control strategies on dengue transmission in Guangzhou. The knowledge generated from this research would provide useful information for authorities regarding the historic patterns of dengue outbreaks, as well as the effectiveness of different disease management strategies.

## Introduction

Dengue is the most prevalent arboviral infection among humans globally [1]. In China, dengue cases have been recorded each year for the past 25 years [2]. Although dengue in China is considered to be non-endemic, a total of 55 114 dengue cases were reported between 2005 and 2014 of which over 85% occurred in 2014 alone. The focus of this study was on Guangzhou (Guangdong Province) in south-east China, as 94% of the recorded dengue cases in 2014 were observed there [2]. Cheng *et al*. highlight the earlier timing of local dengue transmission to be one of the main determinants of the outbreak size in 2014, in particular the increased number of imported cases in May and June of that year. Excess rainfall in 2014 was also reported to have contributed to the outbreak size [3].

The mosquito *Aedes albopictus*, one of the main vectors of dengue, is responsible for most of the dengue transmission in China, including in Guangzhou [4, 5]. Changes in climatic factors can alter the abundance and habitat distribution of different dengue vectors including *Ae. albopictus*. A study by Kearney *et al*. [6] showed how different weather patterns such as warming (increasing temperatures from 0.8 to 1.5 °C) can potentially affect water availability and adult and larval cold tolerance limits of dengue vector mosquitoes, which in turn has an impact on the species abundance and habitat availability as well as the overall mosquito survival. The change in climatic factors is relevant to China because over the past five decades this country has recorded an increase in average temperatures of 1.2 °C [7]. This is estimated to increase even further by 1–5 °C by 2100 and increased precipitation is predicted for the southern parts of China [7].

The incidence of dengue in Guangzhou has the potential to be influenced by changes in weather patterns; it is therefore important to understand the relationship between climatic factors, *Ae. albopictus* abundance and the occurrence of dengue outbreaks. Understanding these patterns in Guangzhou will help to better develop effective public health strategies to combat the potential increases in dengue outbreaks.

Changes in climate and other environmental factors are important for *Ae. albopictus* and therefore the occurrence of dengue outbreaks. Dengue simulation models can help examine the climatic factors that might be important for predicting dengue outbreaks. One particular dengue simulation model that is comprised of two integrated models representing human (DENSiM) and mosquito (CIMSiM) population parameters has been used and validated in Australia, Malaysia [8] and Latin America [9, 10]. Dengue simulation models such as DENSiM allow modelling of current and historic patterns of recorded dengue cases in relation to mosquito population dynamics. DENSiM is coupled to an underlying Container-Inhabiting Mosquito Simulation model (CIMSiM) [11]. CIMSiM simulates container-breeding mosquito populations and DENSiM takes those mosquito populations created in CIMSiM, and overlays them on a specific human population with known age structure, seroprevalence and size. The risk of dengue transmission can then be simulated by introducing dengue viruses (one or multiple serotypes) into DENSiM. These particular models have been used to model dengue transmission in relation to climate change, and other models have been used for similar purposes (e.g. to understand the relationship between surface water and dengue outbreak occurrences [12], and to identify the relationship between *Aedes* mosquitoes, dengue transmission and climatic factors [13]). Although statistical modelling approaches have been applied previously to investigate the relationships between environmental variables and dengue transmission (e.g. 14, 15), to date, such mechanistic simulation models have not been used in China. In this study, we aimed to re-parameterise and validate the CIMSiM/DENSiM dengue simulation models for a large city in China (Guangzhou), where *Ae. albopictus* is the primary dengue vector. We then intend in subsequent work to use this model to test the potential impact of climate change and different control strategies on dengue transmission.

## Methods

### Parameterisation of CIMSiM and DENSiM for Guangzhou

First, CIMSiM [11] was used to simulate mosquito population dynamics in Guangzhou, dependent upon inputs including local meteorological data, mosquito food availability, availability of various breeding sites (containers) and human demographic data [11].

CIMSiM has previously been run using field-validated parameters for the dengue vector *Aedes aegypti* [16]. *Ae. albopictus* coexist with *Ae. aegypti*, but occupy a slightly different ecological niche and are adapted differently to climatic conditions. *Ae. albopictus* is considered the main vector of dengue in Guangzhou [4]; therefore, we recalibrated the CIMSiM model using field data for *Ae. albopictus* for this study.

*Ae. albopictus* population data were collected in Malaysia by the Institute for Medical Research, Kuala Lumpur in 2011 and were provided for use in validating the simulation models for Guangzhou (RH unpubl. data). *Ae. albopictus* are more tolerant to cooler temperatures compared with *Ae. aegypti* [17]; thus, the larval and pupal cold and heat tolerance limits were adjusted in CIMSiM (Table 1). Meteorological data were obtained from the World Weather Local Weather Forecast Asia (http://en.tutiempo.net/) (2015) for the years 2005–2012. These consisted of daily maximum, minimum and average temperatures, daily rainfall and daily humidity values for Guangzhou.

**Table 1.** Temperature thresholds of *Aedes aegypti* compared with *Aedes albopictus*, as used in DENSiM modelling [17]

	*Ae. aegypti*	*Ae. albopictus*
Low-temperature development threshold	16 °C	11 °C
High-temperature development threshold	39 °C	37 °C
Low lethal temperature threshold	12 °C	10 °C
High lethal temperature threshold	43 °C	40 °C

Food delivery rates to mosquito larvae in CIMSiM were iteratively adjusted using the ‘food fitter’ function so that mosquito production (as pupae per container type) matched that of field data used for calibration. The three main containers that were used in the model were buckets, pot plant bases and tyres. Whilst the three container types do not replicate all conceivable larval production sites in Guangzhou, they represent a range of possible container diversity available as well as it shows a range of filling and emptying rates which adequately represent the diversity of the larval environment in the study area.

To re-parameterise DENSiM for Guangzhou, the produced *Ae. albopictus* population outputs from CIMSiM were overlayed on to the estimated human population simulated for Guangzhou in DENSiM, to enable dengue virus transmission to be estimated. A small representative human population of 10 000 was used for the simulations, as this study was seeking to model a representation of a group of people who might reasonably exist together in a discrete community (e.g. in a suburb or district).

Age-specific birth and death rates for China were obtained from the United States Census Bureau [18]. All four possible dengue serotypes were included in the simulation model. The pre-existing herd immunity for the population in this model was set at 0.0542 referring to previously reported seroprevalance values [19].

### Model simulations

China has a comprehensive disease surveillance system that is incorporated into the network of Centres for Disease Control and Prevention (CDC). This includes both provincial and national level surveillance. The majority of the hospitals are directly linked to the CDC infectious disease reporting system [20]. The surveillance system in China for infectious disease such as dengue is mainly hospital-based, and together with the diagnostic laboratories, these form the first reporting line in disease outbreaks [21].

In previous years, infectious disease monitoring and surveillance for diseases such as dengue were underfunded and limited resources were available, specifically in more rural and remote areas. More recently, the laboratory diagnostic methods have been improved. The ‘Chinese Field Epidemiology Training Program’ was established in 2001 which was a training programme to increase the number of trained health professionals to strengthen the surveillance programme, but by 2014, only 194 health care professionals graduated from this programme [20]. There is still no equal access to all of the data reported across the different regional and provincial areas of China, and under-reporting of cases remains a big problem in China, especially in the more remote regions. Due to possible under-reporting or detection of particular serotypes responsible for an outbreak, the accuracy of serotypes circulating may be impacted.

Therefore when modelling dengue transmission, different iterations of serotype introductions were simulated. DENSiM permits up to four different serotypes to be modelled concurrently, and given the history of serotype variation in Guangzhou [22], we initially produced serotype introduction regime that would mimic known serotype detections [22]. In addition, we developed other serotype introduction scenarios to determine which one may provide the best fit to historical dengue incidence and outbreak data.

Four different simulation scenarios were run (Table 2). Scenario 1 involved serotypes to be introduced in different years as not all four serotypes are responsible for the dengue outbreaks each year. Based on previously reported serotype data [22] from 2003 until 2012, only the serotype reported for each year was introduced in the model at the time when it occurred. DENV1 was introduced in 2006 in the model, DENV4 in 2010 and DENV 3 and DENV 2 in 2009. DENV 3 was first detected in 2009, whereas DENV 4 has re-emerged in 2010 after being absent from the region for 20 years [22].

**Table 2.** Four different virus introduction scenarios used in the DENSiM model for the period 2005–12

	Scenario 1	Scenario 2	Scenario 3	Scenario 4
Serotypes introduced	DENV1, DENV2, DENV3, DENV4	DENV1, DENV2, DENV3, DENV4	DENV1, DENV2, DENV3, DENV4	DENV1
First year of introduction	DENV1: 2006 DENV2: 2009 DENV3: 2009 DENV4: 2010	DENV1: 2006 DENV2: 2007 DENV3: 2008 DENV4: 2009	DENV1: 2006 DENV2: 2007 DENV3: 2008 DENV4: 2009	DENV1: 2006
Duration of introduction	10 viraemic persons per week from mid-January onwards for each introduction year	DENV1: January 2006–October 2007 (10 viraemic persons a week) DENV2: January 2007–October 2008 (10 viraemic persons a week) DENV3: January 2008–October 2009 (10 viraemic persons a week) DENV4: January 2009–October 2010 (10 viraemic persons a week)	10 viraemic persons a week from mid-January onwards for each introduction year	10 viraemic persons a week from mid-January onwards for the study period

DENSiM: dengue simulation model; DENV: dengue virus; the introduction time and duration in the model was based on previous studies conducted on serotype circulation during dengue outbreak periods [22].

Scenario 2 involved the introduction of a different serotype in each year of the first 4 years of the model simulation period. Each serotype introduction was modelled to run for a 22 months period to simulate co-circulation of serotypes within the study population as well as to show periods where one serotype is no longer present and another is the more dominant serotype. This type of serotype co-circulation and dominance of one serotype over different periods of time has been demonstrated by different studies [23, 24]. Scenario 3 was run the same as scenario 2 except the serotype introduction periods were continuous. Lastly, scenario 4 involved the introduction of only one serotype in 2006 (DENV1). Different serotype introduction periods in the model were used to account for the co-circulation of the serotypes that do occur in Guangzhou (given the combination of imported and indigenous dengue cases). Additionally the better performance of scenario 4 might be explained by the likelihood that outbreaks in Guangzhou can occur with a single dominant serotype as discussed in the paper by Cheng *et al*. [3] which looked at the large outbreak in 2014, where 98% of dengue cases tested were infected by DENV-1.

The exact number of infected people/travellers coming into Guangzhou is unknown and difficult to estimate. Therefore, a number of iterations of different virus importation rates (number of cases) were tested before a rate of 10 introductions per week was settled upon (Table 2). Ten replications were run for each scenario and the mean number of simulated dengue cases per month was calculated.

### Model outputs

#### Dengue cases

The primary output from the model simulations was the number of prevalent dengue cases per day. Simulated dengue case data from the model output recorded as daily prevalent cases were compared with the reported dengue case data, recorded as number of incident cases per month, for the time period of 2006–2012.

In order to be able to assess model performance meaningfully, the simulated prevalent cases were converted to incident cases. The viraemic duration of an infected person was set to 5 days in DENSiM and the incubation duration to 4 days. That means on each day in the simulated dataset, the recorded infected persons from the previous 4 days have to be removed to be able to report only new (incident) cases of dengue each day. Daily incident cases were totalled for each month to correspond to the recorded dengue case data.

#### Dengue outbreaks

To assess model fit, the simulated monthly dengue case data were recoded to indicate whether an outbreak had occurred or not (1 = outbreak or 0 = no outbreak) for each month of the simulation. To do this, thresholds for consideration of whether an outbreak had occurred were calculated. Three different thresholds were calculated to create three dengue outbreak outcome variables to test which outbreak variable allowed the best model fit [25]. The three thresholds above which an outbreak was considered to have occurred included incident cases exceeding (1) the mean for each month, (2) the mean plus 1.5 s.d. and (3) the mean plus 2 s.d.. When the number of incident dengue cases for a month was below the given threshold, no outbreak was considered to have occurred.

### Assessment of model performance

First, we analysed the validity of the dengue simulation model by using cross-correlation analysis (in Stata, V.14) to compare the simulated case data with the reported dengue cases. Then, the accuracy (sensitivity, specificity, positive and negative predictive value) of each of the four model simulation scenarios was assessed by comparing actual and predicted outbreaks per month. Receiver operator characteristic curves were used to determine the best model fit.

A prediction of high incidence, for example, could lead to a government department using its limited resources to utilise costly measures such as vector control spraying, when a large outbreak may not occur. Therefore, the model with the highest positive predictive value (PPV) was considered for this study to have the best model fit. Previous studies have shown that for dengue management strategies to be effective, a dengue prediction model with high PPV is desirable [26].

### Identification of dengue seasonality

To determine whether dengue outbreaks occur seasonally in Guangzhou, the months were categorised into seasons. In Guangzhou, winter is comprised of December to February, spring is March to May, summer covers June to August and September to November are the autumn months. Simple linear regression was used to test the probability of outbreaks occurring each year in the same season in Guangzhou. The model simulated number of incident cases per month was set as the dependent variable and season was the independent variable. All analyses were undertaken using statistical software STATA (V.14). Statistical significance was considered at *α* = 0.05.

## Results

**Fig. 1.**
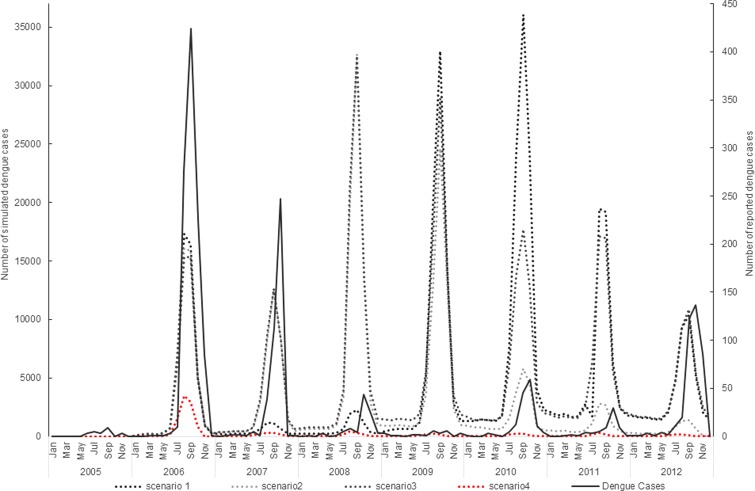
Virus introduction scenarios comparing simulated and reported dengue cases. Four different simulation scenarios were run (Table 2). Scenario 1 involved serotypes to be introduced at different points in the model, DENV1 was introduced in 2006, DENV4 in 2010 and DENV 3 and DENV 2 in 2009. Scenario 2 involved the introduction of a different serotype in each year of the first 4 years of the model simulation period. Each serotype introduction was modelled to run for a 22 months period to simulate co-circulation of serotypes within the study population. Scenario 3 was run the same as scenario 2 except the serotype introduction periods were continuous. Scenario 4 involved the introduction of only one serotype in 2006 (DENV1). The four scenarios are compared with the reported dengue case data.

The simulation model overall appeared to follow a pattern of dengue incidence broadly consistent with reported dengue cases in Guangzhou (Fig. 1).

The simulation model output from DENSiM showed the best fit with incident case data from Guangzhou when only one serotype (scenario 4) was introduced in the virus introduction scenarios, based on cross-correlation results, even though multiple serotypes do exist in nature.

Scenario 4 resulted in a correlation coefficient of 0.75, which reflects the highest correlation between the actual simulated dengue incidences in Guangzhou, out of all four scenarios simulated (Table 3).

**Table 3.** Correlation coefficient for each virus introduction scenario

Virus scenario	*r*
One	0.28
Two	0.37
Three	0.32
Four	0.75

*r*, Correlation coefficient.

**Fig. 2.**
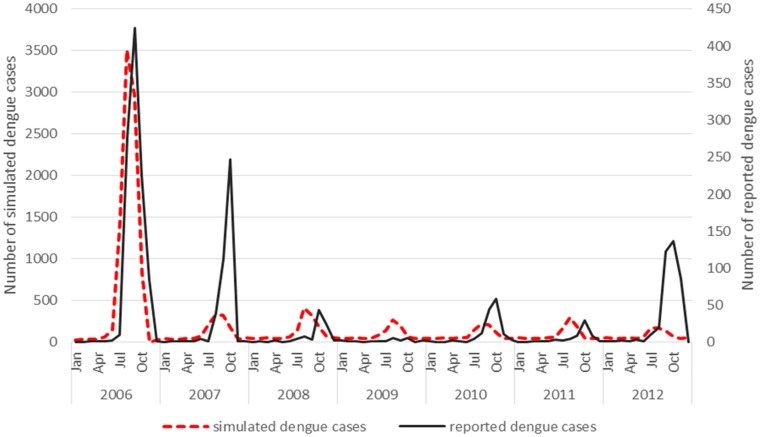
Virus introduction scenario 4 comparing simulated and reported dengue cases. Simulation model run from 2006 to 2012 shows the best fit with incident case data from Guangzhou when only one serotype (scenario 4) is introduced. This was then modelled against the reported dengue cases and resulted in a correlation coefficient of 0.75.

Virus introduction scenario 4 predicted outbreaks with a PPV of 0.83, a sensitivity of 0.18 and a specificity of 0.98 when applying a mean threshold for the outbreak/no outbreak months (Table 4). However, some slight underprediction of cases still occurs in the model in 2007 and 2012 (Fig. 2).

**Table 4.** Sensitivity and specificity of the final model for predicting outbreak presence or absence using different thresholds (using scenario 4)

Threshold	Sensitivity	Specificity	PPV	NPV
Mean	0.18	0.98	0.83	0.74
Mean + 1.5 s.d.	0.18	0.97	0.67	0.80
Mean + 2 s.d.	0.14	0.96	0.4	0.87

s.d., standard deviation; PPV, positive predictive value; NPV, negative predictive value.

The underprediction of the model in year 2007 and 2012 could be explained by the fact that the actual PPV in the field is potentially lowered due to the accuracy of reported cases and the current surveillance system is not capturing all of the serotypes, micro climatic factors that need to be considered that may influence the conditions in the field could also impact on the PPV that the model does not account for. The estimated biting rates (contact between person and mosquito) may be higher in some areas where *Ae. aegypti* coexists with *Ae. albopictus* which is not reflected in the model as *Ae. albopictus* has been reported as the main vector in Guangzhou and therefore only one species has been included in the simulation model. The NPV may therefore be affected in the field as areas where *Ae. aegypti* coexists with *Ae. albopictus* have not been factored into this model. This would have an impact on biting rates and the contact rate between mosquito and human.

A seasonal trend can be observed when the dengue cases are plotted over the study period 2006–2012. Cases tend to occur between July and November (summer and autumn) each year (Fig. 2). The results of the simple linear regression (Table 5) confirmed this, showing that dengue transmission is significantly less likely to occur in spring and winter compared with summer. No significant difference was found between autumn and summer.

**Table 5.** The occurrence of dengue incidences in relationship to the different seasons

Incident cases	Coefficient	CI 95%	*p*-value
Summer	*Reference*		
Autumn	−72.2	−383.0 to 238.5	0.65
Winter	−348.1	−658.9 to −37.4	0.03
Spring	−345.9	−656.7 to −35.1	0.03

CI, confidence interval.

## Discussion

Using the DENSiM dengue model, a working simulation of dengue incidence in Guangzhou was created. The model outputs replicated observed dengue transmission dynamics, albeit with underprediction of dengue activity in 2007 and 2012. The virus introduction scenario, using a single serotype, showed the best model fit to the actual dengue transmission occurring in Guangzhou, even though multiple serotypes do occur in the area and are responsible for different outbreaks each year.

There are several potential reasons why the developed dengue simulation model did not work as well using multiple serotypes in the virus introduction scenarios. First, there is a possible existing bias in the reporting of actual dengue cases which can lead to under-reporting of the magnitude of an outbreak, or even the incidence of dengue in a given time period if cases have not been recorded [27]. Another reason is the set simulated population size which differs from the actual population size, due to computer simulation capacities of the dengue model used. This may influence modelled outbreak size and dynamics. Lastly, a potential issue could be the different localised weather conditions in Guangzhou. The model cannot account for small temperature fluctuations, which also have an influence on the local mosquito population activity profiles in the different areas of the city as microclimates and habitat availability can vary greatly within a large city such as Guangzhou.

The dengue model that has been developed in this study may have worked best with a single serotype introduction as one virus strain can be more prevalent throughout an outbreak over longer periods of time or for a single outbreak period. This was demonstrated in the 2013 outbreak in Yunnan Province where DENV 3 was the main serotype recorded [28].

The study by Sang *et al*. [23] also showed that of all four dengue serotypes being detected in Guangzhou, DENV 1 was the predominant serotype reported, supporting the findings from this study that the best model fit using virus introduction scenario 4 (using just the DENV1 serotype) is a plausible simulation for dengue transmission dynamics in Guangzhou. The simulated dengue transmission in Guangzhou using one serotype only was most successful at demonstrating the reported dengue transmission in the area over the 2006–2012 period. This can possibly be explained by the findings of Sang *et al*. [23] who found that dengue viruses (different serotypes with various genotypes) are likely to be reintroduced to Guangzhou from other countries. The different dengue serotypes, therefore, have not been able to establish an endemic cycle of dengue virus transmission in the area, potentially leading to one serotype to be more dominant in an outbreak period over another even though multiple genotypes have been detected in Guangzhou.

Additionally, the better performance of scenario 4 might be explained by the likelihood that outbreaks in Guangzhou can occur with one more dominant serotype as discussed by Cheng *et al*. [3] when they looked at the large outbreak in 2014, where 98% of cases tested were infected by DENV-1. The poorer performance of the model when multiple serotypes are used could be due to the reported serotypes not reflecting the reality of the field surveillance conducted. Field surveillance may not be sufficient enough to detect all serotypes, this may lead to the model underpredicting cases as well as the modelling being based on an estimated 10 viraemic persons being introduced, this may not be representative enough of the actual number (imported and indigenous cases).

Notwithstanding these limitations, the developed mechanistic simulation model for dengue transmission in Guangzhou displays the seasonal transmission patterns of dengue, highlighting the increased transmission times in summer and autumn. A study by Li *et al*. [29] supports these findings showing that both imported as well as indigenous dengue cases were relatively rare in spring or winter, with increased cases in summer and peak number of cases recorded for autumn.

Even though the dengue transmission model for Guangzhou closely simulated the reported dengue transmission, there are some limitations in the model, such as under-reporting of the dengue outbreak size still occurred in the years 2007 and 2012. Based on some of the difficulties measuring exact outbreak size and dengue importation rates, perfect function of the model is not expected (nor required) for moving forwards to test the different disease control strategies.

This study models outbreak occurrences (dengue cases reported or not) and the accuracy of these can allow the assessment of the different dengue control strategies. Outbreak size is not used as a measure to identify the effectiveness of reducing dengue transmission once the control has been applied. For this purpose, the model is acceptable, having a relatively high PPV of 0.83, meaning outbreak occurrences are well predicted. Not all measures are controllable or accounted for in the simulation scenario. Issues such as under-reporting, inaccuracies in the number of infected travellers and the inability to account for micro climates affecting mosquito population dynamics may affect the model's ability to simulate outbreaks. Overall however, the model reflects dengue transmission dynamics in Guangzhou and may allow the assessment of the effectiveness of dengue control strategies.

This is the first time, based on current knowledge of the literature, that the dengue model CIMSiM–DENSiM has been reparameterised for the mosquito species *Ae. albopictus*, and its first application in China to simulate dengue outbreak occurrences. This is significant and allows the utility of this model to be extended into other contexts, particularly in Asia where *Ae. albopictus* is the predominant dengue vector. This new parameterisation can now be used to evaluate the potential impact of different vector control strategies or increased temperature scenarios (such as under climate change) on dengue transmission in Guangzhou.
